# Impaired TIGIT expression on B cells drives circulating follicular helper T cell expansion in multiple sclerosis

**DOI:** 10.1172/JCI156254

**Published:** 2022-10-17

**Authors:** Hiromitsu Asashima, Pierre-Paul Axisa, Thi Hong Giang Pham, Erin E. Longbrake, William E. Ruff, Nikhil Lele, Inessa Cohen, Khadir Raddassi, Tomokazu S. Sumida, David A. Hafler

**Affiliations:** Departments of Neurology and Immunobiology, Yale School of Medicine, New Haven, Connecticut, USA.

**Keywords:** Autoimmunity, Immunology, Adaptive immunity

## Abstract

B cell depletion in patients with relapsing-remitting multiple sclerosis (RRMS) markedly prevents new MRI-detected lesions and disease activity, suggesting the hypothesis that altered B cell function leads to the activation of T cells driving disease pathogenesis. Here, we performed comprehensive analyses of CD40 ligand– (CD40L-) and IL-21–stimulated memory B cells from patients with MS and healthy age-matched controls, modeling the help of follicular helper T cells (Tfh cells), and found a differential gene expression signature in multiple B cell pathways. Most striking was the impaired TIGIT expression on MS-derived B cells mediated by dysregulation of the transcription factor *TCF4*. Activated circulating Tfh cells (cTfh cells) expressed CD155, the ligand of TIGIT, and TIGIT on B cells revealed their capacity to suppress the proliferation of IL-17–producing cTfh cells via the TIGIT/CD155 axis. Finally, CCR6^+^ cTfh cells were significantly increased in patients with MS, and their frequency was inversely correlated with that of TIGIT^+^ B cells. Together, these data suggest that the dysregulation of negative feedback loops between TIGIT^+^ memory B cells and cTfh cells in MS drives the activated immune system in this disease.

## Introduction

Relapsing-remitting multiple sclerosis (RRMS) is a genetically mediated autoimmune disease mediated by myelin-reactive T cells attacking the CNS (1–3) and is characterized by inflammatory lesions predominantly in the white matter. B cell depletion in patients with RRMS markedly prevents new MRI-detected lesions and disease activity (4), suggesting that altered B cell function leads to the activation of T cells driving disease pathogenesis. Moreover, changes in T cell populations after B cell depletion therapies suggest that the interplay between T cells and B cells is a key feature of the disease pathogenesis (5).

The rapid decrease in disease activity after anti-CD20 antibody treatment despite a lack of major changes in oligoclonality in the cerebrospinal fluid (CSF) implies that antibody-independent B cell functions such as cytokine production and/or the expression of costimulatory or coinhibitory receptors are related to disease pathogenesis (2, 6). Specifically, circulating B cells from patients with MS produce more proinflammatory (IL-6, TNF, GM-CSF) and fewer antiinflammatory (IL-10) cytokines (7), and induction of B cells that enhance IL-10 production have been thought to have potential for clinical application (8–10). Coinhibitory receptors expressed on T cells have a pivotal role in the maintenance of immune homeostasis, and altered expression and function have been linked to autoimmune diseases (11–15). In this regard, Xiao et al. demonstrated that mice lacking T cell immunoreceptor with Ig and ITIM domains (TIGIT) expression in B cells (TIGIT^BKO^ mice) developed severe experimental autoimmune encephalomyelitis (EAE) (16), suggesting the importance of TIGIT on B cells in maintaining CNS tolerance. However, whether TIGIT expression on B cells affects T cell function in MS pathogenesis is not known.

Specific subsets of CD4^+^ Th cells, such as Th1 and Th17 cells, have been suggested to play a critical role in MS pathogenesis (17, 18). In addition, recent data indicate that circulating T follicular helper (cTfh) cells are correlated with the progression of MS disability, and single-cell RNA-Seq (scRNA-Seq) data from human samples and mouse models demonstrated a pathological function of Tfh cells in the disease (19–21). Tfh cells can support B cell differentiation through IL-21 and other cell-surface molecules, while B cells can regulate Tfh functions apart from antibody production (22–24). In the context of these data, we hypothesized that alterations in B cell function could drive the increased activation state of cTfh cells, leading to a hyperactive immune system in patients with MS.

Here, we show that, in patients with MS, memory B cells stimulated in vitro with CD40 ligand (CD40L) and IL-21, modeling the help of Tfh cells, have unique gene expression profiles compared with age-matched, healthy donor–derived memory B cells. Among differentially expressed genes (DEGs), TIGIT expression on MS-derived memory B cells was substantially impaired. Our in vitro experiments demonstrated that *TCF4* was a key transcription factor for TIGIT expression and that *TCF4* expression was dysregulated in patients with MS. Activated cTfh cells expressed CD155, the ligand of TIGIT, and TIGIT on B cells regulated the proliferation of cTfh cells and IL-17 production, independent of IL-10 production. Finally, the proportion of TIGIT^+^ B cells was inversely correlated with the frequency of CCR6^+^ cTfh cells, which was markedly increased in patients with MS. These data suggest that the interaction between TIGIT on activated memory B cells and CD155 on activated Tfh cells is a negative feedback mechanism to suppress the proliferation of Tfh cells, independent of IL-10 production, and that this feedback mechanism is impaired by dysregulation of the *CD40/TCF4/TIGIT* axis in patients with MS.

## Results

### Decreased TIGIT induction in MS-derived memory B cells after CD40L and IL-21 stimulation.

**Figure 1:**
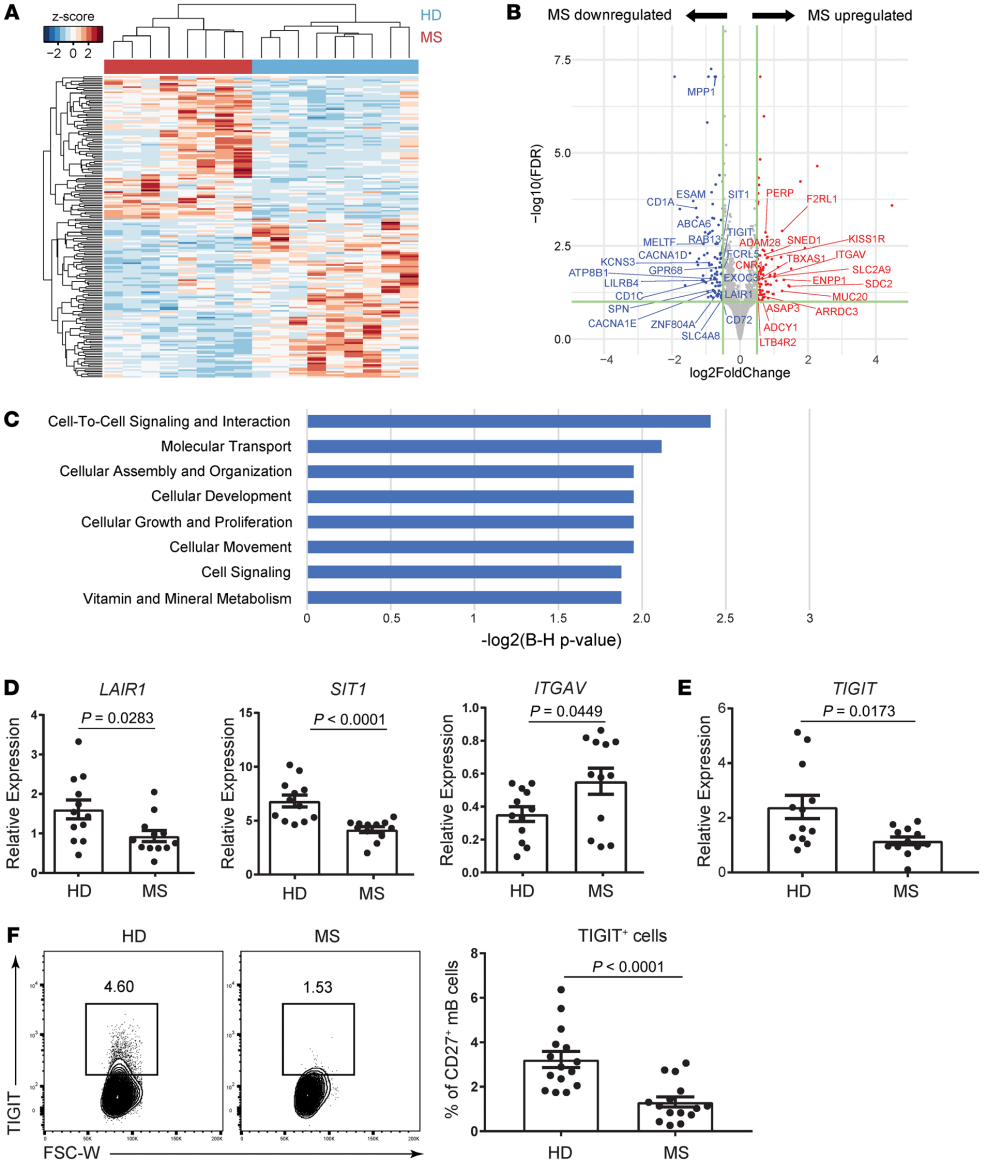
TIGIT is downregulated on memory B cells in MS. (**A**–**C**) Sorted CD20^+^CD27^+^ memory B cells from patients with MS (*n* = 8) and healthy donors (HD) (*n* = 9) were cultured with CD40L and IL-21 for 2 days, and RNA-Seq was performed. (**A**) Heatmap of DEGs (|log_2_ FC| >0.5, FDR < 0.1) in patients with MS and healthy donors. (**B**) Volcano plot depicting DEGs in memory B cells. Red dots represent significantly upregulated genes in MS-derived memory B cells, and blue dots represent significantly downregulated genes. Genes whose location is categorized as “plasma membrane” by IPA software are labeled. (**C**) IPA was performed to identify signatures related to altered molecular and cellular functions. Functions whose –log (Benjamini-Hochberg [B-H] *P* value) values were greater than 1.8 are shown. (**D**–**F**) Sorted CD20^+^CD27^+^ memory B cells from patients with MS and healthy donors (*n* = 12 each) were cultured with CD40L and IL-21 for 2 days. Gene expression was measured relative to *B2M* by qPCR (**D** and **E**). Representative flow data for TIGIT expression (**F**, left) and proportion of TIGIT^+^ cells (**F**, right). mB, memory B cells. Data are presented as the mean ± SEM and were evaluated by 2-tailed, unpaired Student’s *t* test (**D**–**F**). FSC-W, forward scatter width.

We first compared in vitro–stimulated B cells between patients with MS and control individuals by examining mRNA expression levels. Since memory B cells comprise the majority of B cells in CSF (25–27), we focused on conventional CD20^+^CD27^+^ memory B cells. Memory B cells have direct contact with T cells, particularly follicular helper T (Tfh) cells, where they become reactivated (28–30). To model the help of Tfh cells in vitro, CD20^+^CD27^+^ MS patient–derived memory B cells (*n* = 8 patients) and control donor–derived memory B cells (*n* = 9 donors) were stimulated with CD40L and IL-21, and gene expression was evaluated by bulk RNA-Seq analysis. We identified 178 DEGs (|log_2_ fold change [FC]| >0.5, FDR < 0.1) between MS patient– and healthy control–derived memory B cells based on their gene expression profiles (Figure 1A). Compared with the healthy control–derived memory B cells, 84 genes were significantly upregulated and 94 genes were downregulated in memory B cells from patients with MS (Figure 1B). Ingenuity Pathway Analysis (IPA) showed that the difference in gene expression between MS patient and healthy control cells was related to cell/cell signaling pathways (Figure 1C). The expression levels of molecules including *LAIR1*, *SIT1*, and *ITGAV*, which are related to cell-cell interactions, were validated by quantitative PCR (qPCR) (Figure 1D). These data suggest that the activated signatures of memory B cells induced by CD40 and IL-21 signaling were different between patients with MS and healthy control individuals.

Given the important role of TIGIT expression on B cells in maintaining CNS tolerance in murine models (31), we focused on the significant downregulation of TIGIT on MS patient–derived memory B cells (log_2_ FC = –0.54, FDR = 0.01). We validated the decrease in expression of TIGIT by qPCR and flow cytometry (Figure 1, E and F). As *PVR* (also known as CD155) and *NECTIN2* (also known as CD112) both bind the coinhibitory receptor TIGIT and the activating receptor *CD226* (also known as DNAM-1) (14), we examined the expression of CD226 on B cells and found no difference between patients with MS and healthy controls (Supplemental Figure 1A; supplemental material available online with this article; https://doi.org/10.1172/JCI156254DS1). Moreover, no correlations were detected between the proportion of TIGIT^+^ B cells and demographic variables such as sex and age or disease activity and disease duration (Supplemental Figure 1, B–E). Thus, TIGIT expression on activated memory B cells was significantly downregulated in patients with MS, irrespective of background. The impaired signature in patients with MS, together with the regulatory role of TIGIT on B cells in a murine model of disease, suggests that the induction of TIGIT conferred the immunosuppressive signature on B cells.

### TIGIT^+^ B cells are distinct from IL-10–producing B cells.

**Figure 2:**
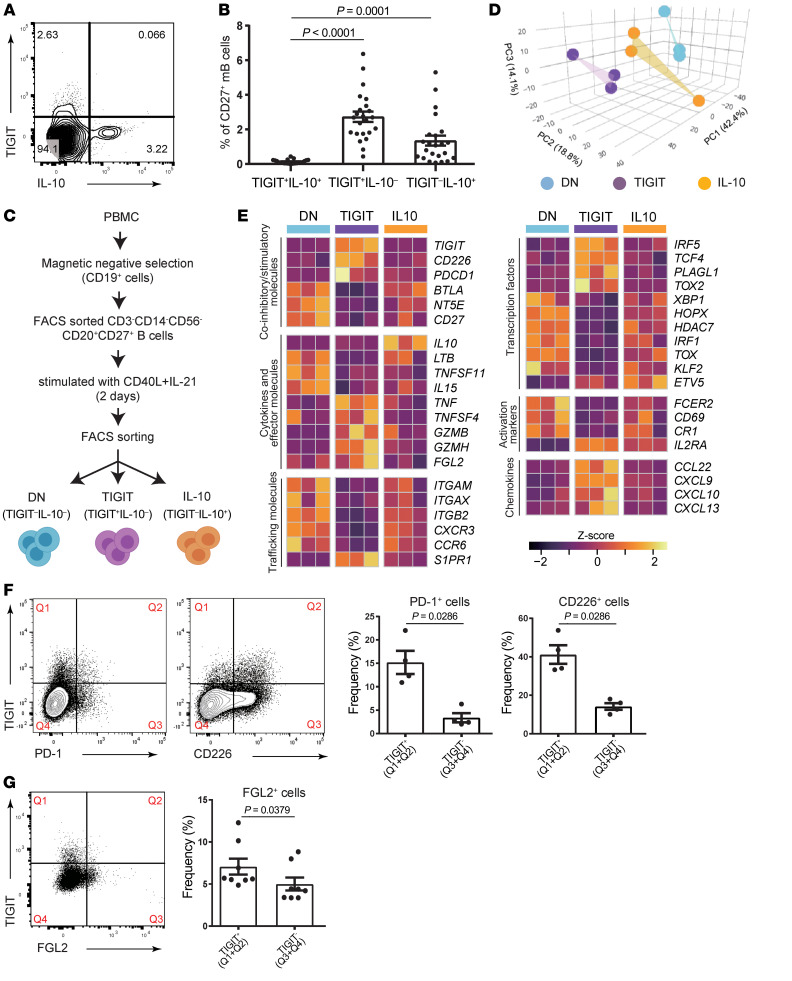
TIGIT is clearly separated from IL-10 expression. (**A**) Representative flow data of TIGIT and IL-10 expression in CD40L+IL-21–stimulated memory B cells. (**B**) The proportions of TIGIT^+^IL-10^+^, TIGIT^+^IL-10^–^, and TIGIT^–^IL-10^+^ (*n* = 23) cells were evaluated by 2-way ANOVA with Dunn’s multiple-comparison test. Data are presented as the mean ± SEM. (**C**) Experimental workflow for RNA-Seq with DN (TIGIT^–^IL-10^–^) cells, TIGIT (TIGIT^+^IL-10^–^) cells, and IL-10 (TIGIT^–^IL-10^+^) cells. (**D**) PCA of RNA-Seq transcriptomes (*n* = 3 healthy donors). (**E**) Heatmap of representative genes that were differentially expressed (|log_2_ FC| >0.5, FDR < 0.1) among 3 groups. (**F**) Representative flow data for PD-1 and CD226 (left) and their frequencies among TIGIT^+^ and TIGIT^–^ cells (right). (**G**) Representative flow data for FGL2 (left) and its frequency among TIGIT^+^ and TIGIT^–^ cells (right). Data are presented as the mean ± SEM. Significance was determined by 2-tailed, unpaired Student’s *t* test (**F** and **G**).

Given that TIGIT contributes to IL-10 production in T cells (14, 32, 33) and TIGIT^+^ human B cells express more IL-10 after CpG (TLR9) stimulation (34), we reasoned that TIGIT expression could be overlapped with IL-10 production on human B cells after CD40L and IL-21 stimulation. To our surprise, the TIGIT^+^ cell population was highly distinct from the IL-10^+^ population, and the frequencies of TIGIT^+^IL-10^+^ B cells were negligible (Figure 2, A and B). To better understand the differences between TIGIT^+^ B cells and IL-10–producing B cells, we analyzed gene expression patterns using RNA-Seq (Figure 2C). Principal component analysis (PCA) placed TIGIT^+^IL-10^–^ (TIGIT^+^) B cells, TIGIT^–^IL-10^+^ (IL-10^+^) B cells, and TIGIT^–^IL-10^–^ (double-negative [DN]) B cells as distinctive cell populations, underlying their unique gene profile (Figure 2D). Of particular interest, the patterns of trafficking molecules, cytokines, and chemokines expressed by TIGIT^+^ B cells were distinct from those of IL-10^+^ B cells and DN B cells (Figure 2E). Moreover, *PDCD1* (also known as PD-1) and *CD226*, but not *BTLA* or *NT5E* (also known as CD73), mRNAs were highly expressed in TIGIT^+^ B cells (Figure 2E). Although not all TIGIT^+^ B cells coexpressed PD-1 or CD226, flow cytometric analysis showed higher expression levels of these molecules in TIGIT^+^ B cells than in TIGIT^–^ B cells (Figure 2F). *HAVCR2* (also known as TIM3) also had a trend toward higher expression in TIGIT^+^ B cells (log_2_ FC = 0.30, FDR = 0.14, compared with DN cells), whereas *HAVCR1* (also known as TIM1) was not detected on B cells, diverging from observations in mice (16). TIGIT^+^ B cells expressed higher *IL2RA* (also known as CD25) but lower *CD69* and *FCER2* (also known as CD23) mRNA levels, indicating that TIGIT^+^ B cells were not simply in a more activated state than the other cell subsets (Figure 2E). Intriguingly, TIGIT^+^ B cells also produced more FGL2 than did TIGIT^–^ B cells, which are known immunosuppressive molecules in Tregs (33) (Figure 2G). In total, these data suggest that the TIGIT^+^ B cells were not related to the IL-10–producing B cells and had a distinct pattern of gene expression.

### Suppressed TIGIT expression in MS is unrelated to plasmablast differentiation program.

We examined the expression of TIGIT and IL-10 in B cells following stimulation with CD40L and IL-21. As previously reported (35), CD27^int^CD38^+^ plasmablasts had a higher capacity to express IL-10 (Supplemental Figure 2, A and B). On the other hand, TIGIT^+^ B cells expressed little CD38 after stimulation, which suggests that TIGIT expression was transient and disappeared after the differentiation of plasmablasts. There were no differences between the proportions of these B cell subsets when comparing cells from patients with MS and healthy controls (21, 36) (Supplemental Figure 2C), and, similarly, no differences were observed in the expression levels of the transcription factors *IRF4*, *PRDM1*, and *XBP1* with regard to the plasmablast developmental program (37, 38) (Supplemental Figure 2D). Thus, the differentiation of plasmablasts suppressed TIGIT expression on B cells, and this signature was unrelated to the downregulation of TIGIT expression on MS-derived B cells.

### Memory B cells have a unique capacity to express TIGIT.

TIGIT expression on the surface membrane was negligible on human B cells, and there were no significant differences in the absolute numbers of ex vivo TIGIT^+^ B cells between healthy controls and patients with MS (Supplemental Figure 3, A and B). Using our scRNA-Seq data sets (25), we also evaluated TIGIT expression on ex vivo B cells at the transcriptional level, and we found no differences between the 2 groups (Supplemental Figure 3, C–E). These data suggest that TIGIT expression is induced only after the activation of human B cells.

We further investigated which B cell subsets can express TIGIT after activation. We sorted B cells into 4 subsets according to CD27 and IgD expression levels and stimulated them with CD40L and IL-21 (Supplemental Figure 4, A and B). Compared with IL-10, which is produced by cells from all the subsets, CD19^+^CD20^+^CD27^–^IgD^+^ naive B cells did not express TIGIT, whereas memory B cells (CD19^+^CD20^+^CD27^+^IgD^+/–^ and CD19^+^CD20^+^CD27^–^IgD^–^) did express TIGIT. Since stimulations affect the polarization of B cell differentiation and activation, we also activated B cells with CpG or anti-IgM antibodies (B cell receptor [BCR]) (Supplemental Figure 4C). CD40L-stimulated B cells expressed significantly higher levels of TIGIT than did B cells under other stimulation conditions, implying that activation through CD40 favored TIGIT upregulation.

### IL-4 suppresses TIGIT expression.

**Figure 3:**
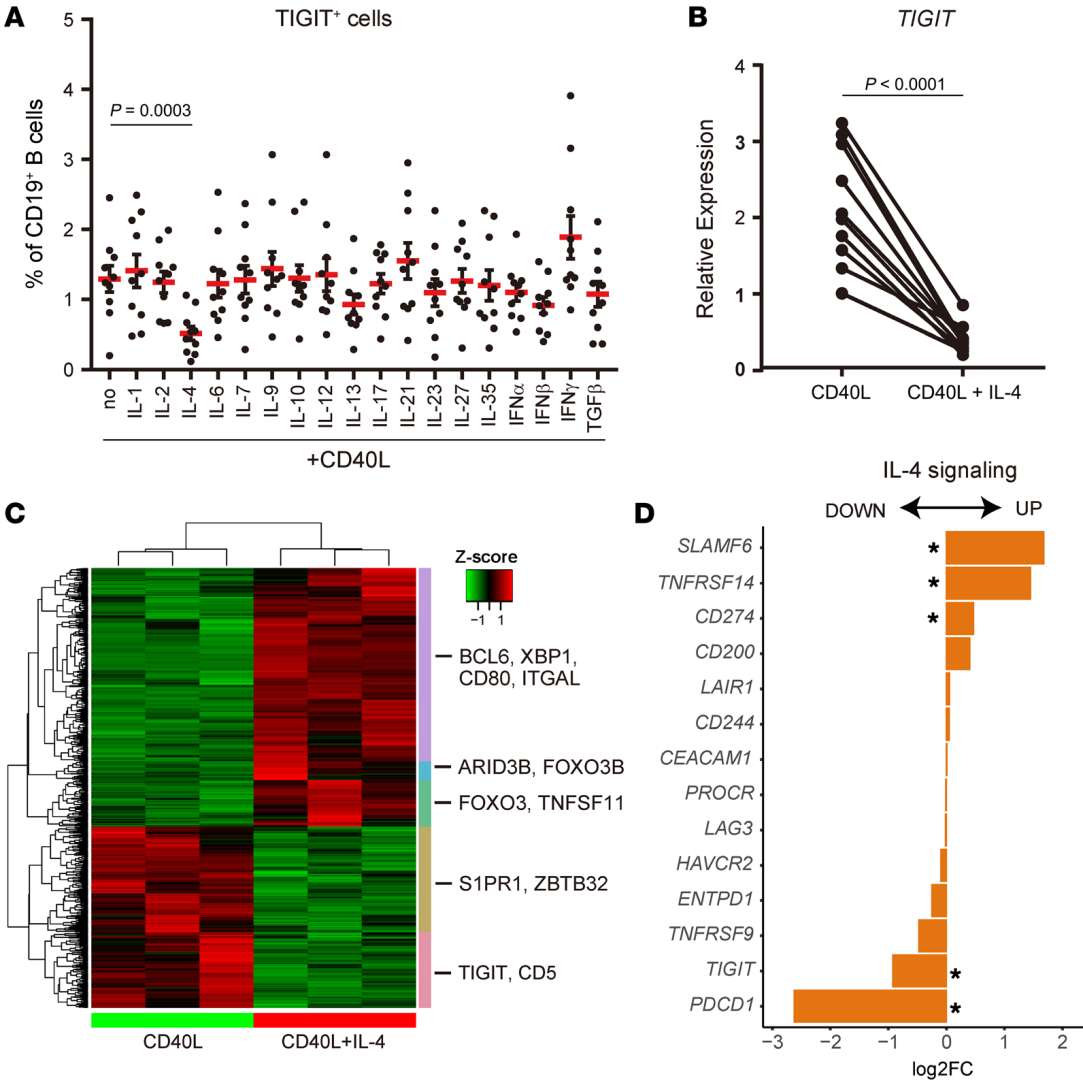
IL-4 treatment suppresses TIGIT expression on B cells. (**A**) Healthy donor–derived CD19^+^ B cells (*n* = 10) were stimulated with CD40L in the presence of the indicated cytokines. The frequencies of TIGIT^+^ cells were measured by flow cytometry. Data are presented as the mean ± SEM and were evaluated by Dunnett’s multiple-comparison test. (**B**) Sorted CD20^+^CD27^+^ memory B cells from healthy donors were cultured with CD40L or CD40L+IL-4 for 2 days, and *TIGIT* mRNA expression was measured relative to *B2M* by qPCR (*n* = 11). Data were evaluated by 2-tailed, unpaired Student’s *t* test. (**C** and **D**) Sorted CD20^+^CD27^+^ memory B cells from healthy donors (*n* = 3) were cultured with CD40L or CD40L+IL-4 for 2 days, and RNA-Seq was performed. Heatmap of DEGs (|log_2_ FC| >0.5, FDR < 0.1; 736 genes) between CD40L and CD40L+IL-4 conditions. (**C**) Representative genes are depicted. (**D**) Coinhibitory receptor expression pattern in CD40L or CD40L+IL-4–stimulated memory B cells. *FDR < 0.1.

We evaluated the effects of various cytokines in modifying TIGIT expression. IL-4, a key cytokine signal for B cell activation and differentiation, significantly downregulated TIGIT expression (Figure 3, A and B). We then examined the gene expression profile of B cells in the presence or absence of IL-4 by performing RNA-Seq and identified 736 DEGs between memory B cells stimulated with CD40L alone and memory B cells stimulated with both CD40L and IL-4 (CD40L+IL-4) (Figure 3C). We examined other coinhibitory/stimulatory receptors and observed that IL-4 induced *SLAMF6*, *TNFRSF14*, and *CD274* expression, but inhibited *PDCD1* expression on memory B cells (Figure 3D). These data demonstrate that IL-4 could control the expression of multiple coinhibitory and stimulatory receptors with significant suppression of TIGIT expression.

### TCF4 is a key transcription factor for TIGIT expression on B cells.

**Figure 4:**
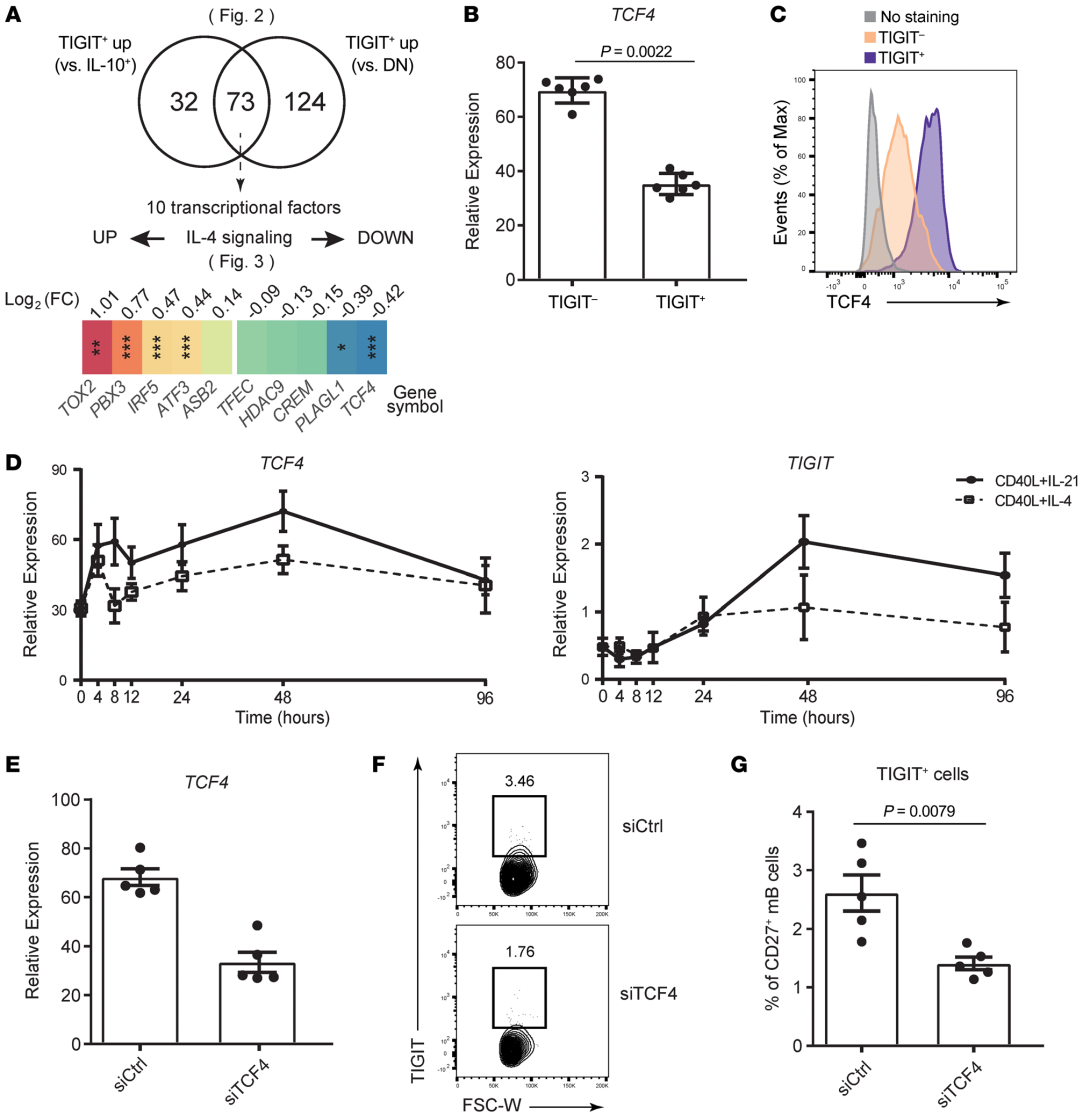
*TCF4* induces TIGIT expression on memory B cells. (**A**) Venn diagrams showing the overlapped genes. Significantly upregulated genes (log_2_ FC >0.5, FDR < 0.1) in TIGIT^+^IL-10^–^ cells compared with TIGIT^–^IL-10^+^ cells and TIGIT^–^IL-10^–^ cells were evaluated. Among the 73 overlapped genes, transcription factors are highlighted, and heatmaps are depicted on basis of the log_2_ FC under the IL-4–stimulated condition. *FDR < 0.1, **FDR < 0.01, and ***FDR < 0.001. (**B**) TIGIT^+^ and TIGIT^–^ cells were sorted from CD20^+^CD27^+^ memory B cells stimulated with CD40L+IL-21 for 2 days, and *TCF4* mRNA expression was measured relative to *B2M* by qPCR (*n* = 6). Significance was determined by 2-tailed, unpaired Student’s *t* test. (**C**) Representative histogram of TCF4 expression by flow cytometric analysis of TIGIT^+^ cells, TIGIT^–^ cells, and control (no staining). Max, maximum. (**D**) mRNA expression kinetics of *TCF4* and *TIGIT* from 7 different time points (*n* = 7). Data are presented as the mean ± SEM. (**E**–**G**) CD20^+^CD27^+^ memory B cells were transfected with an siRNA targeting TCF4 (siTCF4) or the control (siCtrl). *TCF4* expression was measured relative to *B2M* by qPCR, and 51% knockdown efficiency was confirmed (**E**). Representative flow data for TIGIT expression (**F**) and the proportion of TIGIT^+^ cells (**G**). Data are presented as the mean ± SEM and were evaluated by 2-tailed, unpaired Student’s *t* test (**E** and **G**).

To uncover the mechanism of TIGIT expression on B cells, we studied key transcription factors related to TIGIT regulation (Figure 4A). We identified 73 genes that were significantly upregulated in TIGIT^+^ B cells compared with IL-10^+^ B cells and DN (TIGIT^–^IL-10^–^) B cells. Ten genes were categorized as transcription factors, and among them, we focused on *TCF4* (also known as E2-2), as IL-4 treatment significantly downregulated its expression in concordance with TIGIT and resulted in high *TCF4* expression levels in TIGIT^+^ B cells (Figure 4, B and C). We examined the kinetics of *TCF4* gene expression by qPCR and found that its expression was suppressed by IL-4 treatment from the early (4-hour) time point. In light of the TIGIT expression kinetics with later induction, the temporal change in *TCF4* levels could explain its role as an upstream regulator of TIGIT (48–96 hours) (Figure 4D).

To clarify the relationship between *TCF4* and TIGIT, we treated primary memory B cells with an siRNA targeting *TCF4* and evaluated TIGIT expression (Figure 4, E and F). We achieved an approximately 50% knockdown of *TCF4* gene expression (Figure 4E), and this significantly downregulated the expression of TIGIT (Figure 4, F and G). Moreover, we evaluated these signatures using Farage cells, a human B cell line (39) that expresses TIGIT ex vivo (Supplemental Figure 5A). TIGIT expression was upregulated when cells were stimulated with CD40L, while IL-4 treatment suppressed TIGIT expression (Supplemental Figure 5, B–E). These data suggest that Farage cells have the same regulatory mechanisms of TIGIT expression as primary B cells and are useful for our analyses. We found that *TCF4* knockout using CRISPR/Cas9 systems suppressed TIGIT expression (Supplemental Figure 5, F and G). Overall, these data showed that *TCF4*, downstream of CD40 signaling, induced TIGIT expression on human B cells.

### The CD40/TCF4/TIGIT axis is dysregulated in memory B cells in patients with MS.

**Figure 5:**
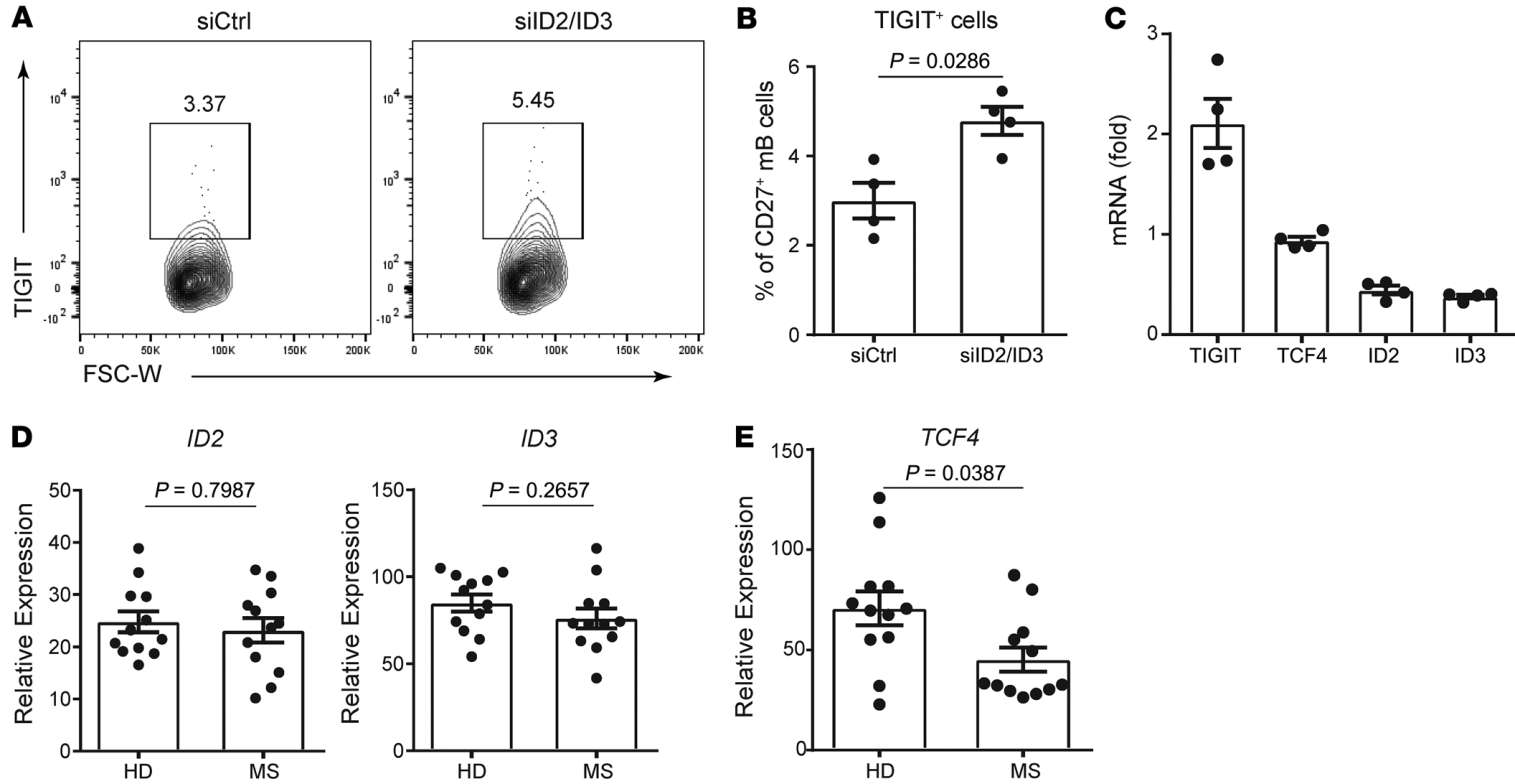
The *CD40/TCF4/TIGIT* axis is dysregulated on MS-derived memory B cells. (**A**–**C**) CD20^+^CD27^+^ memory B cells were transfected with siRNAs targeting ID2 and ID3 (siID2/ID3) or with siCtrl (*n* = 4). (**A**) Representative flow data for TIGIT expression. (**B**) Proportion of TIGIT^+^ cells by flow cytometric analysis. (**C**) *TIGIT*, *TCF4*, *ID2*, and *ID3* expression levels were measured relative to *B2M* by qPCR (FC versus the siRNA/siXBP1 condition). (**D** and **E**) Sorted CD20^+^CD27^+^ memory B cells from healthy donors (*n* = 12) and patients with MS (*n* = 12) were cultured with CD40L+IL-21 for 2 days, and *ID2*, *ID3* (**D**), and *TCF4* (**E**) expression levels were measured relative to *B2M* by qPCR. All data are presented as the mean ± SEM and were evaluated by 2-tailed, unpaired Student’s *t* test (**B**, **D**, and **E**).

Inhibitors of DNA binding and cell differentiation (ID) proteins heterodimerize with basic helix-loop-helix transcription factors such as *TCF4* and negatively regulate activity (40–42). To further investigate the relationship between *TCF4* and TIGIT expression levels, we treated memory B cells with an siRNA targeting both *ID2* and *ID3* and evaluated gene signatures (Figure 5, A–C). Downregulation of both *ID2* and *ID3* substantially upregulated TIGIT expression without changing *TCF4* expression levels. These data support our results showing that *TCF4* is important for TIGIT expression on memory B cells. Although we detected no difference in expression levels of *ID2* and *ID3* between patients with MS and healthy controls, *TCF4* expression was significantly downregulated on MS-derived memory B cells (Figure 5, D and E). Thus, in total, we found that the *CD40/TCF4/TIGIT* axis on memory B cells was dysregulated in patients with MS.

### TIGIT on B cells suppresses the proliferation of CCR6^+^ Tfh cells.

**Figure 6:**
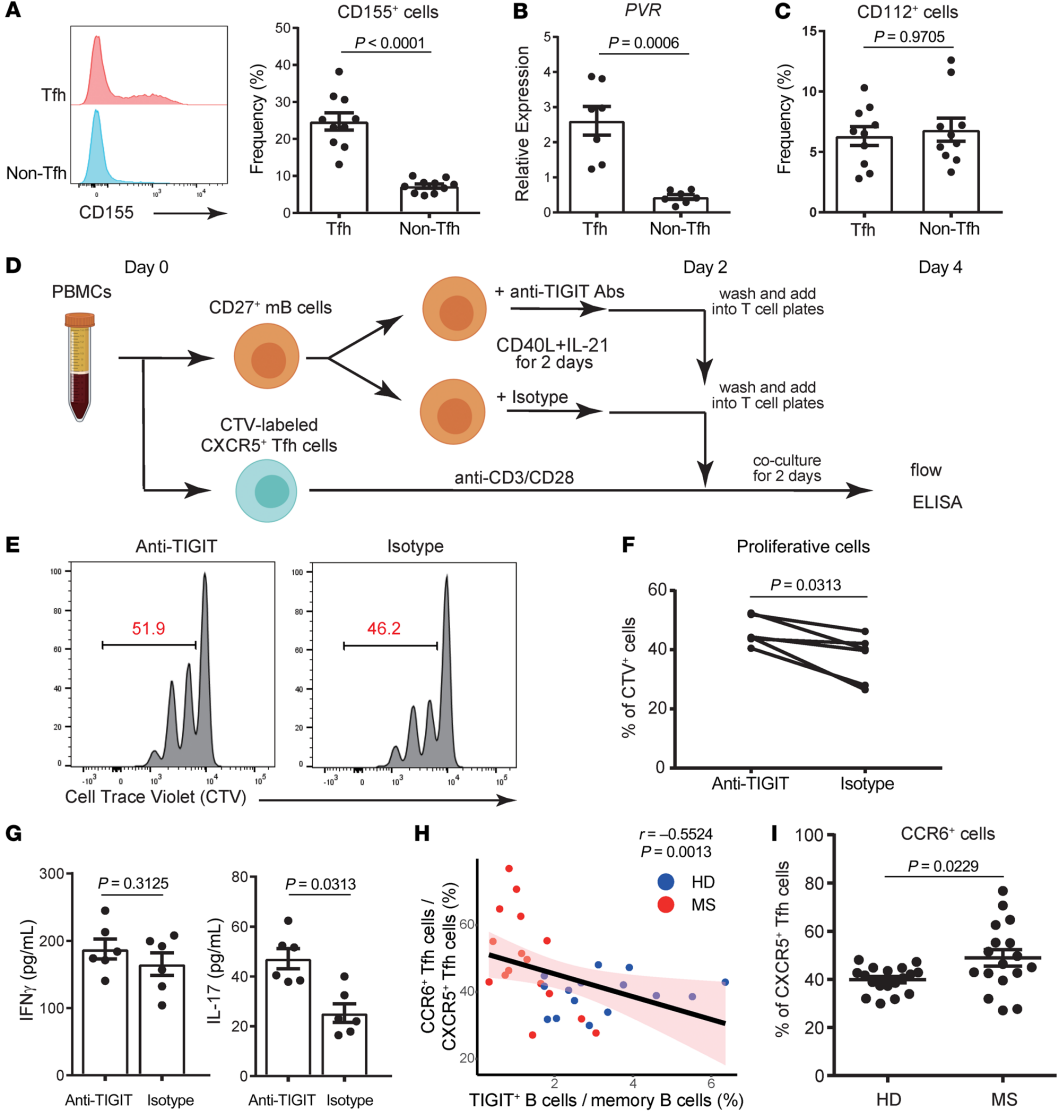
TIGIT^+^ B cells suppress the proliferation of CCR6^+^ Tfh cells. (**A**–**C**) Sorted CD4^+^CD45RA^–^CXCR5^+^ Tfh cells and CD4^+^CD45RA^–^CXCR5^–^ non-Tfh cells were stimulated with anti-CD3/anti-CD28 antibodies (each 1 μg/mL) for 3 days. Representative flow data for CD155 expression (**A**, left) and the proportion of CD155^+^ cells (**A**, right) (*n* = 10) are shown. (**B**) *PVR* mRNA expression was measured relative to *B2M* by qPCR (*n* = 7). (**C**) Proportion of CD112^+^ cells (*n* = 10). (**D**) Experimental workflow for coculture assays with sorted CD20^+^CD27^+^ memory B cells and CD4^+^CD45RA^–^CXCR5^+^ Tfh cells. (**E**) Representative flow data for Tfh cell proliferation. (**F**) Proportion of proliferated CellTrace Violet^+^ (CTV^+^) cells. (**G**) IL-17 and IFN-γ expression in the supernatants of coculture assays was evaluated by ELISA. (**H**) Correlation between CD4^+^CD45RA^–^CXCR5^+^CCR6^+^ Tfh cells (percentage of CD4^+^CD45RA^–^CXCR5^+^ Tfh cells) and TIGIT^+^ cells (percentage of CD20^+^CD27^+^ memory B cells). Data for healthy donors are indicated by blue dots (*n* = 15) and by red dots for patients with MS (*n* = 16). Linear regression is shown with a 95% CI (pink area). (**I**) Proportion of CCR6^+^ Tfh cells between healthy donors (*n* = 18) and patients with MS (*n* = 17). Data are presented as the mean ± SEM and were evaluated by 2-tailed, unpaired Student’s *t* test (**A**–**C** and **I**) or Wilcoxon matched-pairs, signed-rank test (**F** and **G**).

CD40L, also known as CD154, is predominantly expressed on CD4^+^ T cells, and CD40-CD154 interactions facilitate T cell–dependent B cell activation (43, 44). The importance of CD40 signaling for TIGIT expression on B cells led us to hypothesize that TIGIT ligation would drive T cell function. Thus, we evaluated the expression of the TIGIT ligands CD155 and CD112 on CD4^+^CD45RA^–^ memory T cells. Although both molecules were scarcely detectable on T cells ex vivo (data not shown), CD4^+^CD45RA^–^CXCR5^+^ cTfh cells had significantly higher CD155 expression than did CD4^+^CD45RA^–^CXCR5^–^ non-cTfh cells with anti-CD3/anti-CD28 stimulation (Figure 6, A and B). Furthermore, we observed that CD4^+^CD45RA^–^CXCR5^+^CD127^hi^CD25^lo^ effector cTfh cells expressed higher levels of CD155 as compared with CD4^+^CD45RA^–^CXCR5^+^CD127^lo^CD25^hi^ regulatory Tfh cells (Supplemental Figure 6, A and B). There were no differences in CD112 expression between the 2 subsets of T cells (Figure 6C). CD155 expression on T cells mediates costimulatory TIGIT signaling, inducing tolerance and the subsequent suppression of cytokine production (45, 46). To evaluate whether TIGIT expression on activated B cells affected the proliferation of cTfh cells through the TIGIT/CD155 axis, we cocultured memory B cells and cTfh cells with anti-TIGIT antibody (34). TIGIT is also detected in T cells, and to clarify the function of TIGIT on B cells, memory B cells were stimulated with CD40L and IL-21 in combination with anti-TIGIT antibody or an isotype control, washed, and cocultured with CXCR5^+^ cTfh cells (Figure 6D). Intriguingly, we found that TIGIT expression on B cells suppressed the proliferation of cTfh cells (Figure 6, E and F). Moreover, IL-17 production from T cells significantly increased when TIGIT expression was blocked, and the supernatant from coculture assays also showed increased IL-17 secretion (Figure 6G and Supplemental Figure 7, A and B). To further evaluate the direct function of TIGIT on B cells, we cocultured CXCR5^+^ cTfh cells with B cells in which the *TIGIT* gene was deleted by an siRNA and observed the same trend (Supplemental Figure 7, C–E). These data suggest that memory B cells suppress the proliferation of cTfh cells, especially IL-17–producing cTfh cells, through the interaction between TIGIT on B cells and CD155 on cTfh cells.

Finally, we investigated the relationship between TIGIT^+^ B cells and CCR6^+^ cTfh cells, known as IL-17–producing cTfh cells (47). We found that the proportion of TIGIT^+^ B cells was inversely correlated with the proportion of CCR6^+^ cTfh cells (Figure 6H). Moreover, the proportion of CCR6^+^ cTfh cells significantly increased in patients with MS (Figure 6I). These findings support our hypothesis that TIGIT^+^ B cells could suppress the proliferation of predominantly CCR6^+^ cTfh cells and that impairment of TIGIT expression on B cells alters the distribution of cTfh cells in patients with MS (Supplemental Figure 8).

## Discussion

T-B cell interactions play a central role in adaptive immune responses and are highly relevant to autoimmune disease physiopathology. Here, we performed comprehensive analyses of B cells from healthy age-matched controls compared with MS patient–derived memory B cells after stimulation with CD40L and IL-21 in vitro and found a differential gene signature in multiple B cell pathways. Most striking was the impaired TIGIT expression on MS patient–derived B cells mediated by dysregulation of the transcription factor *TCF4*. Our assessment of TIGIT^+^ B cells revealed their capacity to suppress the proliferation of IL-17–producing cTfh cells. Additionally, we found an inverse correlation between the frequency of TIGIT^+^ B cells and that of CCR6^+^ cTfh cells, which was increased in patients with MS. Together, these data suggest that the dysregulation of negative feedback loops between TIGIT^+^ memory B cells and cTfh cells in MS is one of the drivers of immune system activation in this disease (Supplemental Figure 8).

As screening of gene expression profiles of blood B cells ex vivo between patients with MS and controls from our scRNA-Seq data sets did not reveal differences (25), we hypothesized that perturbing the system in vitro rather than investigating the ex vivo steady state was necessary to better model the in vivo system. We found that memory B cells stimulated with CD40L and IL-21, modeling the help of Tfh cells, could allow us to distinguish between patients and controls. It was of interest that TIGIT expression was substantially downregulated after stimulation on memory B cells derived from patients with MS and that TIGIT-expressing B cells were IL-10^–^. While recent studies showed that TIGIT^+^ B cells produced more IL-10 after CpG stimulation (34), we found that TIGIT^+^ B cells and IL-10^+^ B cells clustered separately by PCA with RNA-Seq analysis. Moreover, as shown in Supplemental Figure 2A, although CD38^+^ plasmablasts have a higher capacity to express IL-10, TIGIT^+^ B cells expressed little CD38, which suggests that TIGIT expression disappears after B cells are differentiated into CD38^+^ plasmablasts.

Our RNA-Seq data revealed a unique gene profile of TIGIT^+^ B cells. Among coinhibitory receptors, programmed cell death 1 (PD-1) was found to be expressed on TIGIT^+^ B cells and had the same behavior as TIGIT expression under IL-4 stimulation. Intriguingly, PD-1^+^ B cells also possess a regulatory capacity toward a T cell response via the PD-1/PD-L1 pathway (48). Moreover, TIGIT^+^ B cells produced more fibrinogen-like 2 (FGL2), which has the capacity to suppress T cell proliferation (33, 49). While we demonstrated the regulatory function of TIGIT^+^ B cells on cTfh cells via TIGIT/CD155 pathways, TIGIT^+^ B cells might have additional regulatory functions through various signals.

We examined the transcriptional regulation of TIGIT and observed a role for *TCF4* in regulating TIGIT expression in B cells, which were downregulated in patients with MS. This is of interest, as there are elevated levels of phosphorylated NF-κB after CD40 stimulation in MS-derived B cells (50), and the *CD40* risk variant is related to the lower expression of CD40, affecting impaired B cell functions (1, 51, 52). Moreover, CD40 signaling in B cells is affected not only by genetic factors, but also by vitamin D3 levels, one of the environmental risk factors in MS. Although further investigation is needed, these observations suggest that the dysregulation of CD40 signaling is one of the critical signatures in MS-derived B cells linked to TIGIT expression.

We demonstrated that TIGIT expression on B cells could significantly suppress the proliferation of cTfh cells and IL-17 production in vitro. Moreover, the proportion of CCR6^+^ cTfh cells was markedly increased in the circulation of patients with MS. Consistent with our data, TIGIT^BKO^ mice showed not only the development of more severe EAE, but also an increase in activated CD4^+^ T cells and IL-17 production (16). Furthermore, multimodal single-cell profiling of blood and spatial transcriptomes of brain tissues demonstrated Th17-like Tfh cells in the brains of patients with progressive MS (53). Together with the inverse correlation between TIGIT^+^ B cells and CCR6^+^ cTfh cells, we found that the impaired expression of TIGIT on activated B cells drove the expansion of Th17-like cTfh cells, suggesting a dysregulated loop leading to continued immune activation in patients with MS.

Although we performed a bidirectional analysis of T cells and B cells, we could not evaluate the direct interaction between TIGIT^+^ B cells and Tfh cells in the lymphoid tissue. The activation of Tfh cells is known to be critical for the reactivation of memory B cells, and intravital 2-photon microscopy has shown a direct contact not only at the T-B cell border but also at subcapsular proliferative foci (43, 54, 55). These proliferative foci represent a large surface that extends along the floor of the subcapsular sinus and predominantly consists of B cells, Tfh cells, and CD169^+^ macrophages. Intriguingly, migration into the subcapsular sinus intrinsically requires S1PR1 expression, which is characteristic of TIGIT^+^ B cells (Figure 2E), and scRNA-Seq analysis of mouse lymph nodes showed TIGIT expression on subcapsular proliferative foci–residing B cells (55). Moreover CCR6, one of the characteristic surface markers of IL-17–producing cTfh cells, is important for the accumulation of these cells near the subcapsular proliferative foci (56). Future studies are needed to elucidate the precise relationships between memory B cells and cTfh cells in human lymph nodes by spatial transcriptomics such as DBiT-Seq (57).

In summary, our data implicate a negative feedback loop between memory B cells and CCR6^+^ cTfh cells via TIGIT-CD155 interactions. These investigations provide a potential framework for assessing how immune responses converge after activation and how their dysregulation leads to the development of autoimmune diseases. Moreover, these data shed light on the coinhibitory receptors on B cells and provide potential insights into the antibody-independent functions of B cells in immune-mediated diseases with a possible contribution of aberrant T-B cell interactions.

## Methods

### Study participants

Peripheral blood was drawn from healthy individuals and patients with MS after informed consent was provided. The patients were diagnosed with RRMS according to the 2010 McDonald Criteria and had not been treated with any immunomodulatory therapies in the preceding 3 months at the time of the blood draw. The characteristics of the patients with MS in this study are listed in Supplemental Tables 1 and 2.

### Human B and T cell isolation

PBMCs were isolated from donors by Lymphoprep (STEMCELL Technologies) gradient centrifugation. Total CD19^+^ B cells were isolated by negative magnetic selection using a Human B Cell Isolation Kit (STEMCELL Technologies). In the B and T cell coculture experiments, CD3^+^ cells were first isolated using the Release Human CD3 Positive Selection Kit (STEMCELL Technologies).

### Flow cytometry and cell sorting

For surface staining, single-cell suspensions were prepared from PBMCs and stained with fixable viability dye for 10 minutes at room temperature, followed by staining with surface antibodies for 30 minutes at 4°C. To measure absolute cell numbers, CountBright Absolute Counting Beads (Thermo Fisher Scientific) were used. For cytokine staining, GolgiStop (BD Bioscience) was added for the last 6 hours to B cells, and T cells were stimulated with PMA (50 ng/mL) and ionomycin (1,000 ng/mL) in the presence of GolgiStop (BD Biosciences) for 6 hours. Cells were fixed and permeabilized with the Cytofix/Cytoperm intracellular staining kit (BD Bioscience) for 30 minutes at 4°C, followed by staining for 30 minutes at 4°C. For FGL2 staining, the rabbit monoclonal antibody (catalog PA5-71472; Invitrogen, Thermo Fisher Scientific) was prepared with the conjugation of the Zenon Rabbit IgG Labeling Kit (Thermo Fisher Scientific). For the staining of TCF4, cells were fixed and permeabilized with the Foxp3 Fix/Perm buffer set (eBioscience) for 45 minutes at 4°C, followed by staining with TCF4 rabbit monoclonal antibody (catalog ab217668, NCI-R-159-6; Abcam) or an equivalent amount of normal rabbit IgG as a negative control (catalog ab172730, EPR25A; abcam) for 45 minutes at 4°C. After incubation with the primary antibodies, cells were washed and incubated for 30 minutes at 4°C with anti–rabbit IgG (catalog 406421, Poly4064; BioLegend) as a secondary antibody. Stained samples were analyzed with an LSR Fortessa flow cytometer (BD Biosciences). Data were analyzed with FlowJo software (Tree Star, version 10.4.2). The following antibodies and their clones were used: anti-CD2 (catalog 300222, RPA2.10), anti-CD4 (catalog 317410/317444, OKT4), anti-CD14 (catalog 325604, HCD14), anti-CD19 (catalog 302234/302256, HIB19), anti-CD20 (catalog 302340, 2H7), anti-CD27 (catalog 356418, M-T271), anti-CD38 (catalog 303504, HIT2), anti-CD56 (catalog 304604, MEM-188), anti–IL-10 (catalog 501404, JES3-9D7), anti-CD112 (catalog 337114, TX31), anti-CD155 (catalog 337610/337614, SKII.4), anti–IFN-γ (catalog 506504, B27), anti–IL-17A (catalog 512322, BL168), anti–PD-1 (catalog 329906, EH12.2H7), and anti-CD226 (catalog 338304, 11A8) (all from BioLegend); anti-CD3 (catalog 555332, UCHT1), anti-CD45RA (catalog 560674, HI100), anti-CXCR5 (catalog 558113, RF8B2), anti-CD25 (catalog 562442, M-A251), and anti-CD127 (catalog 560549, HIL-7R-M21) (all from BD Biosciences); and anti-TIGIT (catalog 51-9500-42, MBSA43) (eBioScience).

### Cell culture

#### B cells.

After the isolation of CD19^+^ B cells from PBMCs using the Human B cell Isolation Kit as described above, CD20^+^CD27^+/–^IgD^+/–^ B cells were sorted on a FACSAria (BD Biosciences) and stimulated with CD40L (0.1 μg/mL) (Enzo) and IL-21 (20 ng/mL) (R&D Systems) for 2 days in RPMI 1640 medium (Gibco, Thermo Fisher Scientific) supplemented with 10% FBS, 2 nM l-glutamine, 100 U/mL penicillin, and 100 μg/mL streptomycin (Lonza) using 96-well round-bottomed plates (Corning).

#### T cells.

After the isolation of CD3^+^ T cells from PBMCs using the Human T cell Isolation Kit as above, CD4^+^CD45RA^–^CXCR5^+/–^ T cells were sorted and cultured in the above media. Ninety-six-well round-bottomed plates (Corning) were precoated with anti–human CD3 (catalog 555329, UCHT1) (1 μg/mL) and cultured for 3 days with soluble anti–human CD28 (catalog 555725, CD28.2) (1 μg/mL) (both from BD Biosciences). The culture medium was the same as described above.

### T and B cell cocultures

Sorted CD4^+^CD45RA^–^CXCR5^+^ cTfh cells were stimulated with anti-CD3/anti-CD28 antibodies (each 0.5 μg/mL) for total 4 days. Sorted autologous CD20^+^CD27^+^ memory B cells were stimulated with CD40L (0.1 μg/mL) plus IL-21 (20 ng/mL) and anti-TIGIT antibodies (catalog 15-9500-82, MBSA43) or an equivalent amount of mouse IgG1 kappa isotype control (catalog 16-4714-82, P3.6.2.8.1) (all from eBioScience). After 2 days, B cells were washed twice and cocultured with cTfh cells at a ratio of 1:1 for 2 days. In some experiments, nucleofected B cells with siRNAs instead of anti-TIGIT antibodies were used to knock down TIGIT expression. T cells were labeled using a CellTrace Violet Proliferation Kit (Invitrogen, Thermo Fisher Scientific), and their proliferation was determined on a BD LSR Fortessa flow cytometer. Supernatants were collected, and IFN-γ and IL-17 production was measured by ELISA (R&D Systems).

### qPCR

Total RNA was extracted using an RNeasy Micro Kit (QIAGEN) or a ZR-96 Quick-RNA Kit (Zymo Research) according to the manufacturer’s instructions. cDNA was synthesized with TaqMan Reverse Transcription Reagents (Applied Biosystems) or SuperScript IV VILO Master Mix (Invitrogen, Thermo Fisher Scientific). cDNAs were amplified with TaqMan probes (TaqMan Gene Expression Arrays) and TaqMan Fast Advanced Master Mix on a StepOne Real-Time PCR System (Applied Biosystems) according to the manufacturer’s instructions. RNA expression was measured relative to *B2M* expression. The adopted TaqMan probes were as follows: *B2M* (Hs00187842_m1), *TIGIT* (Hs00545087_m1), *CD226* (Hs00170832_m1), *PVR* (Hs00197846_m1), *NECTIN2* (Hs01071562_m1), *LAIR1* (Hs00253790_m1), *SIT1* (Hs00183946_m1), *ITGAV* (Hs00233808_m1), *XBP1* (Hs00231936_m1), *PRDM1* (Hs00153357_m1), *IRF4* (Hs00180031_m1), *IL10* (Hs00961622_m1), *TCF4* (Hs00162613_m1), *ID2* (Hs04187239_m1), and *ID3* (Hs00171409_m1).

### siRNA knockdown

CD20^+^CD27^+^ memory B cells were sorted after the isolation of CD19^+^ B cells from PBMCs, which were collected from leukopacks (New York Blood Center) and centrifuged at 100*g* for 10 minutes at room temperature. Remove supernatant completely and resuspend the cell pellets with P3 Primary Cell 4D-Nucleofector X solution (Lonza). The final concentration of cells used for nucleofection was 1 × 10^6^ cells/20 μL. Memory B cells were nucleofected with 300 nM siRNAs using the Amaxa 4D Nucleofector system’s program “E0-117” for primary human B cells (Lonza). Immediately after nucleofection, 80 μL prewarmed culture media (same as above) were added to the cuvette, and cells were rested in the incubator for 30 minutes at 37°C. After resting, 20 μL was transferred from the cuvette to 96-well round-bottomed wells with 180 μL prewarmed culture media and a total of 5 wells (2 × 10^5^ cells/200 μL) per 1 siRNA were set. Cells were stimulated with CD40L (0.1 μg/mL) (Enzo) and IL-21 (20 ng/mL) (R&D Systems) for 2 days and further analyses were performed. The adopted siRNAs (all from Dharmacon) used were as follows: ON-TARGETplus Human TCF4 (6925) siRNA, ON-TARGETplus Human ID2 (3398) siRNA, ON-TARGETplus Human ID3 siRNA (3399), ON-TARGETplus Human XBP1 (7494) siRNA, ON-TARGETplus Human TIGIT (201633) siRNA, and ON-TARGETplus Non-targeting Pool (control).

### CRISPR gene knockout

Assembly of ribonucleoproteins (RNPs) was performed as previously described (58). Briefly, chemically synthesized CRISPR-targeting RNA (crRNA) (Dharmacon) and *trans*-activating crRNA (tracrRNA) (Dharmacon) at concentrations of 160 μM were mixed and incubated at 37°C for 30 minutes to form guide RNA (gRNA) at a concentration of 80 uM. This gRNA was mixed at a 1:1 ratio by volume with 40 μM *Streptococcus pyogenes* Cas9 (UC Berkeley QB3 MacroLab) and incubated at 37°C for 15 minutes to form RNPs at a concentration of 20 μM. Farage cells (American Type Culture Collection [ATCC]; CRL-2630) were pooled and centrifuged at 200*g* for 8 minutes at room temperature. The supernatant was removed completely, and the cell pellets were resuspended in SG 4D-Nucleofector X solution (Lonza). The final concentration of cells used for nucleofection was 4 × 10^5^ cells/20 μL. Cells were nucleofected with 4 μL RNPs using the program “CA-137” for B cell lines of the Amaxa 4D Nucleofector system (Lonza). Immediately after nucleofection, 80 μL prewarmed culture media (same as above) were added to the cuvette, and cells were rested in the incubator for 30 minutes at 37°C. After resting, 20 μL was transferred from the cuvette into 96-well round-bottomed wells with 180 μL prewarmed culture media, and a total of 5 wells (0.8 × 10^5^ cells/200 μL) per RNP were set. Cells were cultured for 2 days, and further analyses were performed. The adopted crRNAs (Dharmacon) were as follows: Edit-R Human Synthetic *TCF4* (6925) sgRNA and Edit-R Modified Synthetic crRNA (Scramble; 5′-GGTTCTTGACTACCGTAAT-3′).

### RNA-Seq

#### Preparation of cells.

For the analysis of differences between B cells derived from patients with MS and B cells from healthy donors, CD19^+^ B cells were isolated from PBMCs using the Human B Cell Isolation Kit, and CD20^+^CD27^+^ memory B cells were harvested after the stimulation with CD40L (0.1 μg/mL) plus IL-21 (20 ng/mL) for 2 days. Samples were collected from 8 patients from RRMS and 9 healthy donors. For the analysis of TIGIT expression on B cells, CD3^–^CD14^–^CD56^–^CD20^+^CD27^+^ memory B cells were sorted and stimulated with CD40L (0.1 μg/mL) plus IL-21 (20 ng/mL). After 2 days, TIGIT^–^IL-10^–^ B cells, TIGIT^+^IL-10^–^ B cells, and TIGIT^–^IL-10^+^ B cells were sorted using an IL-10 detection assay (Miltenyi Biotec). CD20^+^CD27^+^ memory B cells were also stimulated with CD40L (0.1 μg/mL) or CD40L (0.1 μg/mL) plus IL-4 (10 ng/mL) for 2 days and harvested. Samples were collected from 3 healthy donors.

#### cDNA and library preparation and sequencing.

RNA was isolated using the RNeasy Plus Micro Kit (QIAGEN), and cDNAs were generated using the SMART-Seq, version 4, Ultra Low Input RNA Kit for sequencing (Takara/Clontech). Barcoded libraries were generated using the Nextera XT DNA Library Preparation kit (Illumina) and sequenced with a 2 × 100 bp paired-end protocol on the HiSeq 4000 or NovaSeq 6000 Sequencing System (Illumina).

#### RNA-Seq data analysis.

Low-quality ends (Phred score <30) and short read length (minimum length = 30) was trimmed using PRINSEQ++ (version 1.2) (59). Trimmed reads were aligned to the hg38 genome reference using STAR (version 2.7.1) (60), and subsequently, RSEM (61) was used to count reads mapping to the genes from Ensembl release 93. For the removal of unwanted variation, the top 5000 genes ranked by edgeR (62) *P* values were set as “in silico empirical” negative controls, and RUVSeq (Bioconductor) (63) was performed. Pair-wise differential expression was analyzed using the R package Deseq2 (64). The cutoff value to select DEGs is provided in each figure legend. Significantly upregulated and downregulated genes from RNA-Seq analyses are shown in Supplemental Table 3.

#### Data availability.

RNA-Seq data were deposited in the NCBI’s Gene Expression Omnibus (GEO) database (GEO GSE211358).

### scRNA-Seq

A PBMC scRNA-Seq data set that we had previously generated (25) was reanalyzed. Gene-cell matrices were analyzed using the Seurat (65, 66) package in R (version 3.6.2), including data integration, clustering, multiplet identification, and cell type annotation. The top 2000 variable genes were selected, and integration anchors were determined by “FindIntegrationAnchors.” These anchors were used to integrate the data using the “IntegrateData” function with the top 30 dimensions and scaled. The top 10 principal components (PCs) were used for data integration and downstream steps, along with a clustering resolution of 0.7. Cluster-specific gene expression profiles were established using the “FindAllMarkers” per cluster and per subset to annotate the clusters. Doublet clusters were determined by coexpression of heterogeneous lineage markers (e.g., *MS4A1* and *CD3*), and these clusters were removed prior to finalizing the UMAPs.

### Statistics

All statistical analyses were performed using GraphPad Prism 7 (GraphPad Software). Detailed information about statistical analysis, including tests and values, is provided in the figure legends. *P* values of less than 0.05 were considered significant.

### Study approval

This study was approved by the IRB of Yale University (2000027291REG). All experiments conformed to the principles set out in the WMA Declaration of Helsinki and the Department of Health and Human Services Belmont Report. The study participants provided informed consent.

## Author contributions

The experiments were conceptualized by HA, TSS, and DAH. HA performed the experiments, analyzed data, and wrote the original draft. PPA, THGP, NL, WER, IC, KR, and EEL helped with sample collection, experiments, and data analyses. TSS and DAH supervised the experiments. All authors reviewed the manuscript.

## Supplementary Material

Supplemental data

Supplemental table 1

Supplemental table 2

Supplemental table 3

## Version 1 10/17/2022

Electronic publication
