# Supplementary material for: Impaired TIGIT expression on B cells drives circulating follicular helper T cell expansion in multiple sclerosis
Source: J Clin Invest. 2022 Oct 17;132(20):e156254. doi: 10.1172/JCI156254 (PMC9566906; doi:10.1172/JCI156254)

Supplemental Figure 1

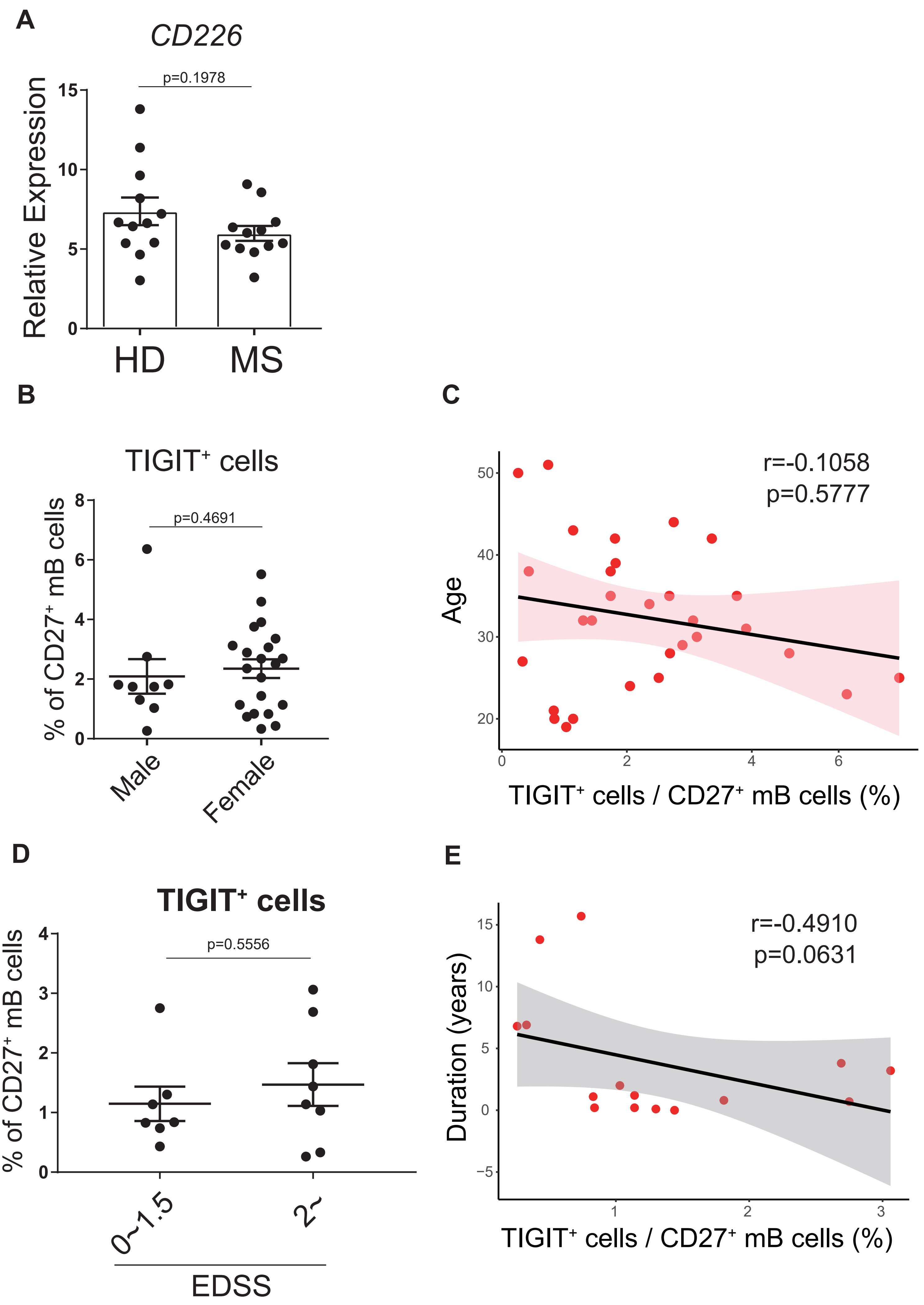

Supplemental Figure 2

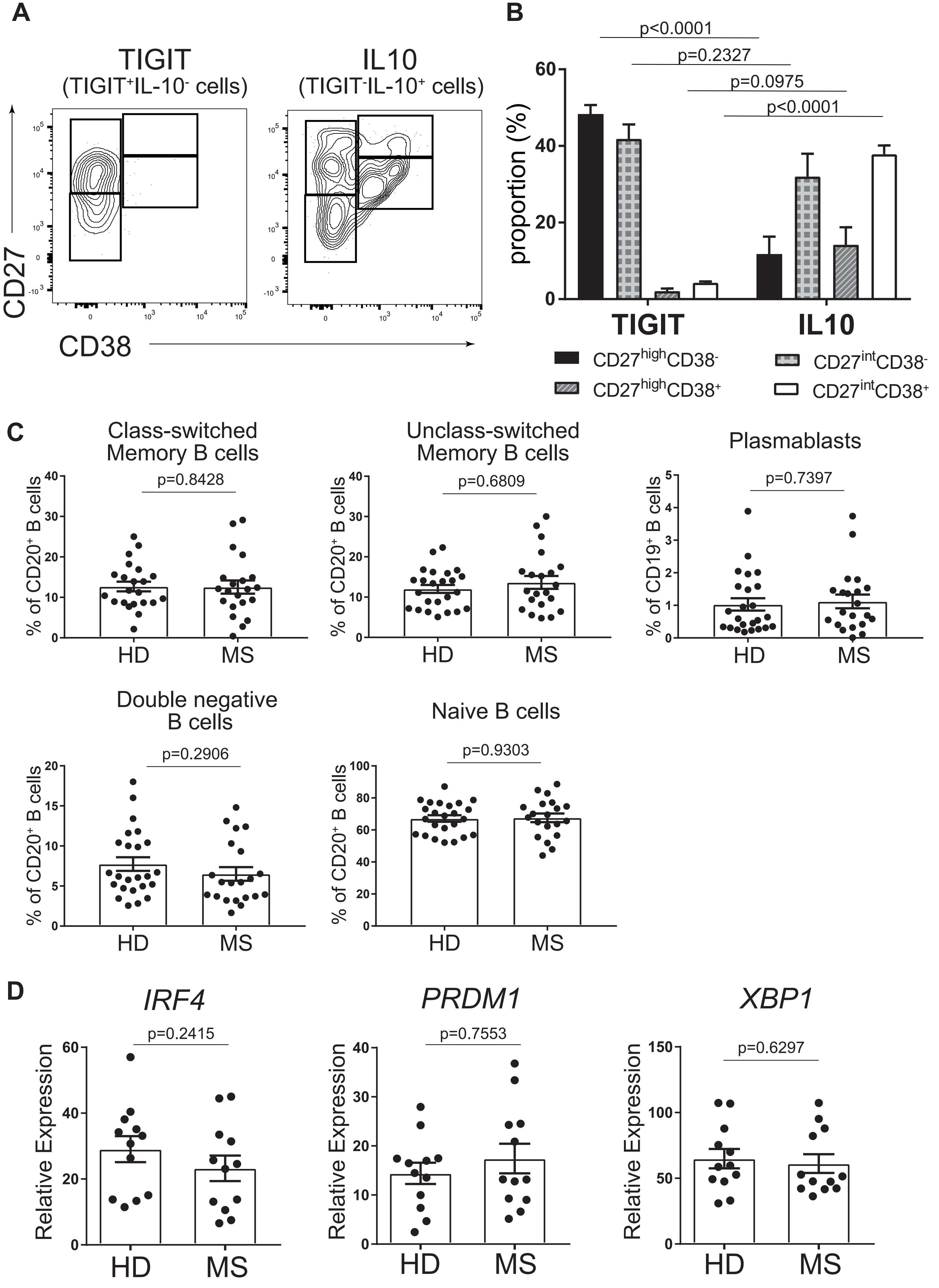

Supplemental Figure 3

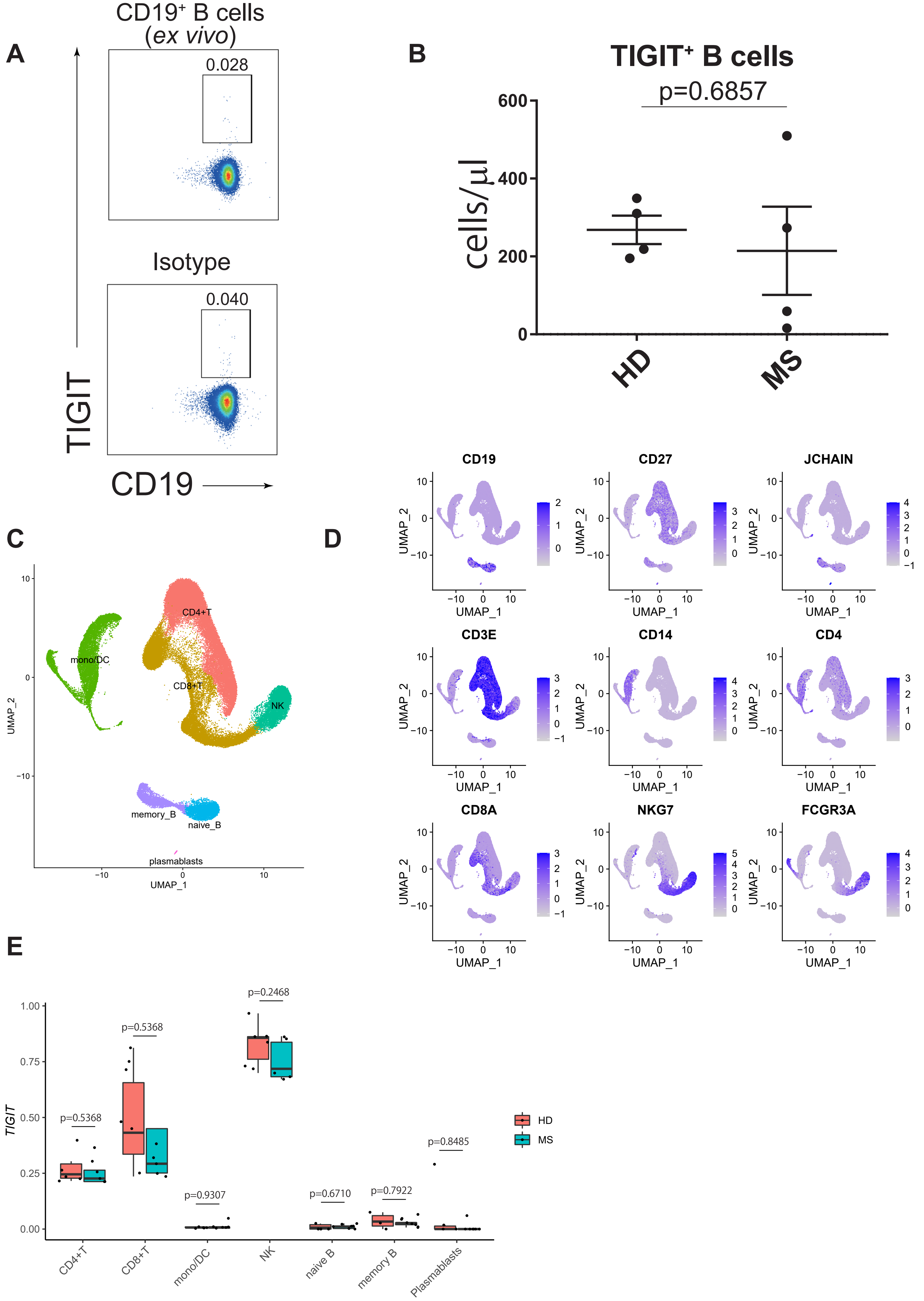

Supplemental Figure 4

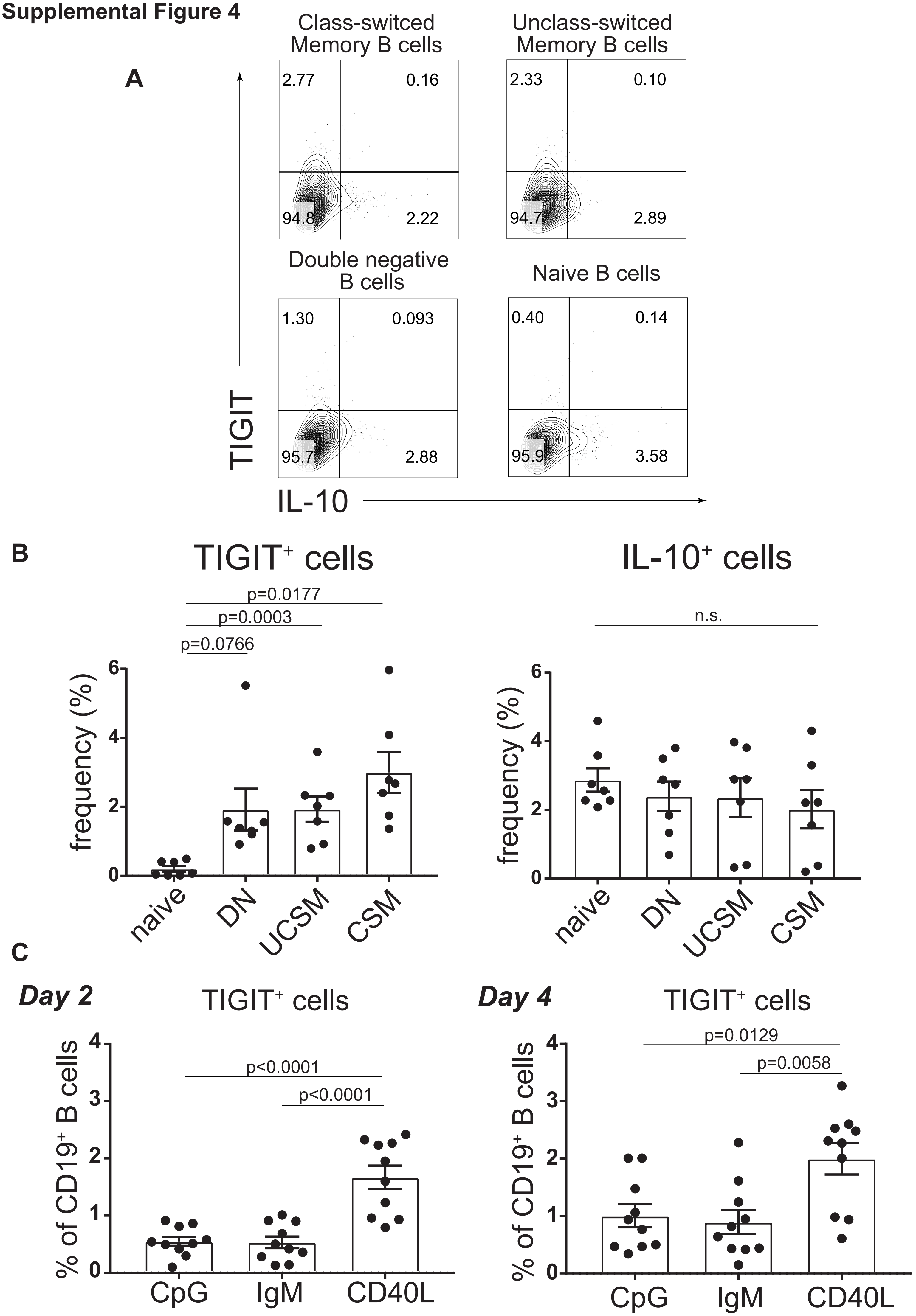

Supplemental Figure 5

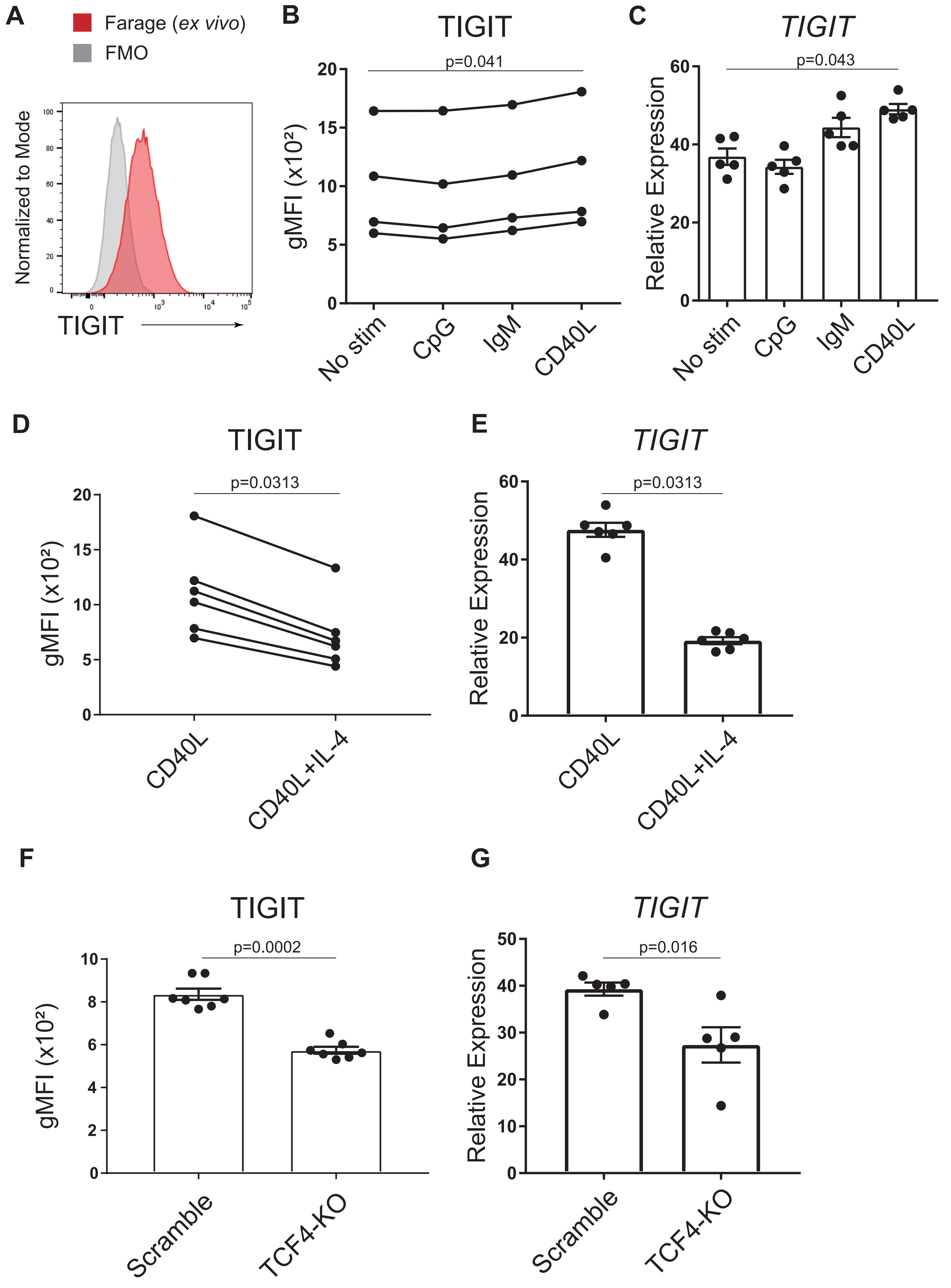

Supplemental Figure 6

A B

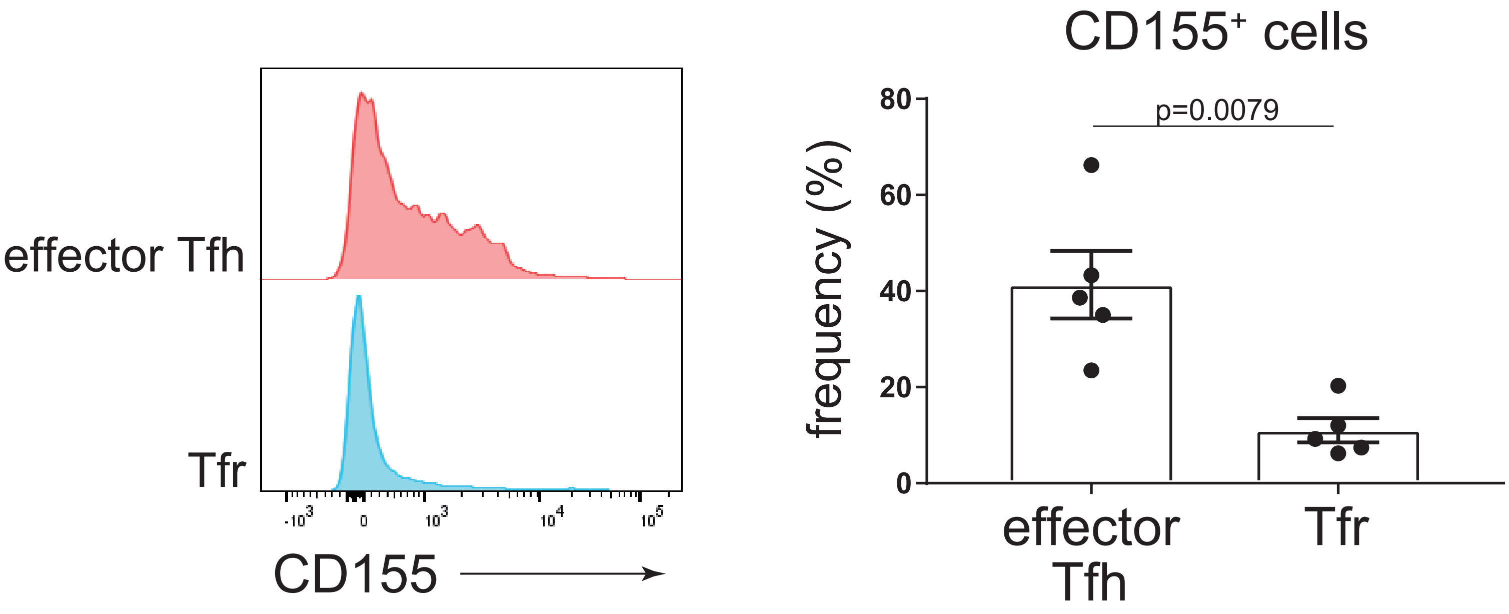

Supplemental Figure 7

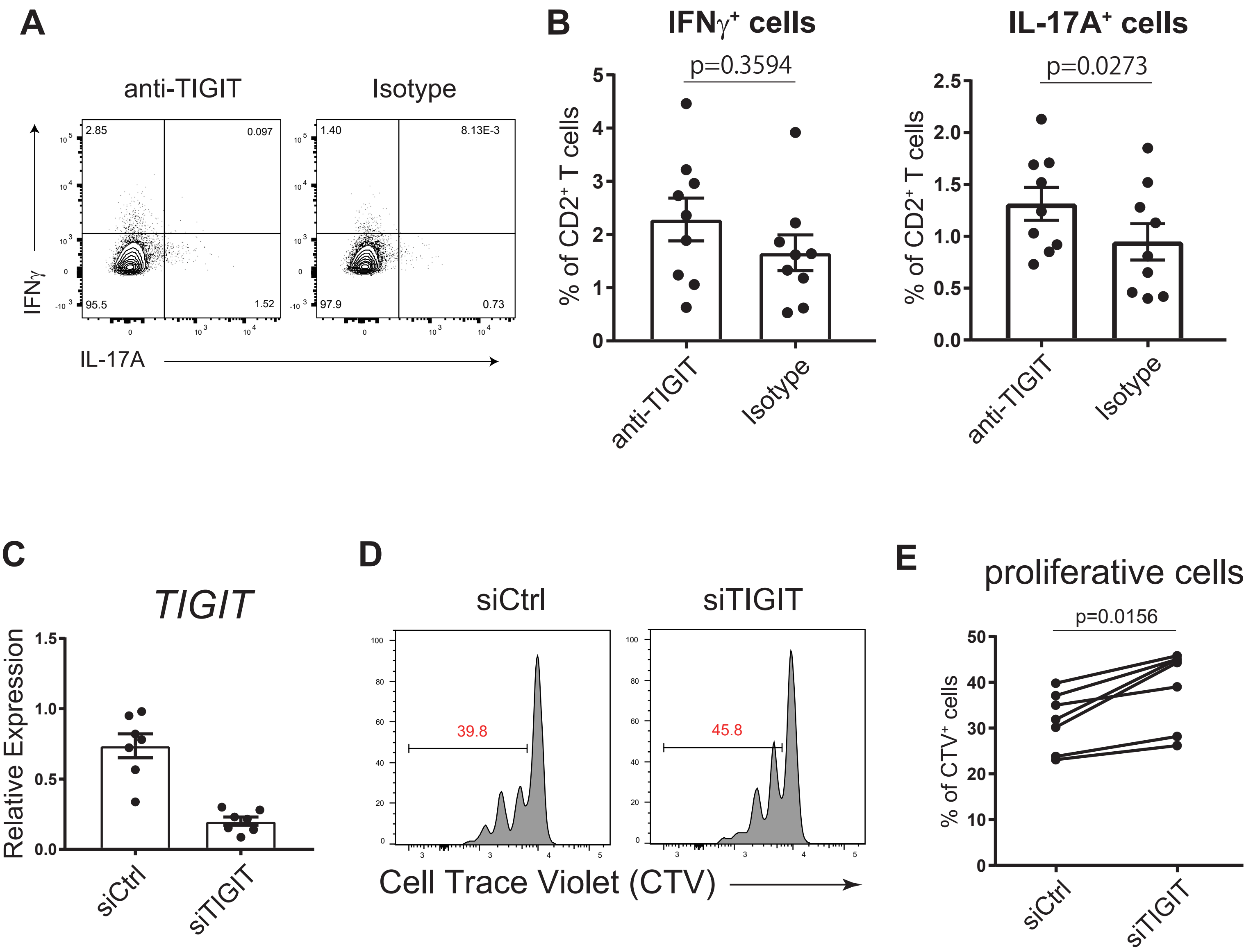

Supplemental Figure 8

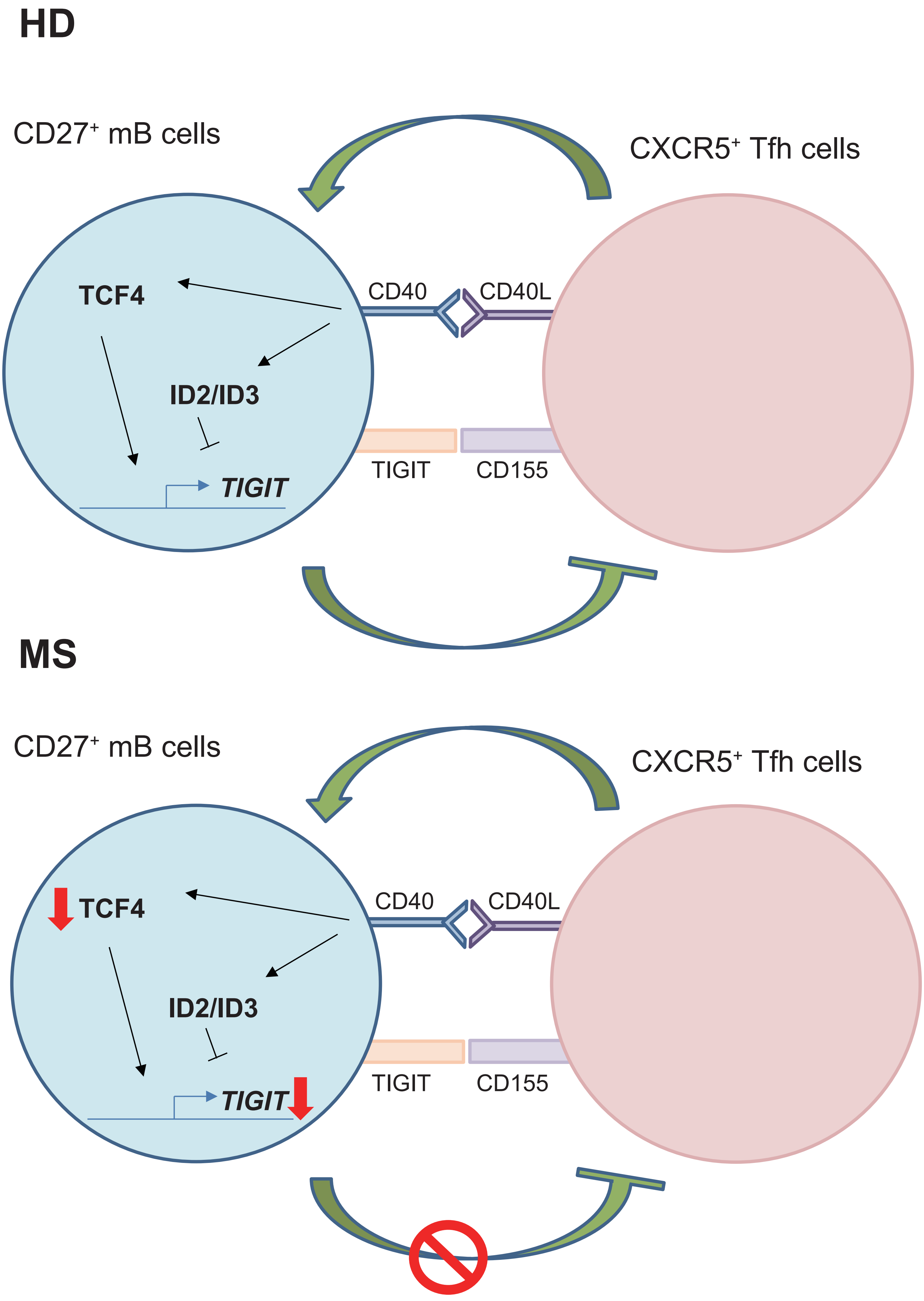

Supplement: Supplemental data [file jci-132-156254-s121.pdf]
